# Exploring ITM2A as a new potential target for brain delivery

**DOI:** 10.1186/s12987-022-00321-3

**Published:** 2022-03-21

**Authors:** Céline Cegarra, C. Chaves, C. Déon, T. M. Do, B. Dumas, A. Frenzel, P. Kuhn, V. Roudieres, J. C. Guillemot, D. Lesuisse

**Affiliations:** 1 Rare and Neurologic Diseases Research Therapeutic Area, Sanofi, Chilly Mazarin, France; 2 Proteomics, Translational Sciences, Sanofi, Chilly Mazarin, France; 3 Sanofi Biological Research, Sanofi, Vitry-Sur-Seine, France; 4 Yumab GmBH, Braunschweig, Germany

**Keywords:** Blood brain barrier, ITM2A, Antibodies, Transcytosis

## Abstract

**Background:**

Integral membrane protein 2A (ITM2A) is a transmembrane protein expressed in a variety of tissues; little is known about its function, particularly in the brain. *ITM2A* was found to be highly enriched in human brain versus peripheral endothelial cells by transcriptomic and proteomic studies conducted within the European Collaboration on the Optimization of Macromolecular Pharmaceutical (COMPACT) Innovative Medicines Initiative (IMI) consortium. Here, we report the work that was undertaken to determine whether ITM2A could represent a potential target for delivering drugs to the brain.

**Methods:**

A series of *ITM2A* constructs, cell lines and specific anti-human and mouse ITM2A antibodies were generated. Binding and internalization studies in Human Embryonic Kidney 293 (HEK293) cells overexpressing ITM2A and in brain microvascular endothelial cells from mouse and non-human primate (NHP) were performed with these tools. The best ITM2A antibody was evaluated in an in vitro human blood brain barrier (BBB) model and in an in vivo mouse pharmacokinetic study to investigate its ability to cross the BBB.

**Results:**

Antibodies specifically recognizing extracellular parts of ITM2A or tags inserted in its extracellular domain showed selective binding and uptake in ITM2A-overexpressing cells. However, despite high RNA expression in mouse and human microvessels, the ITM2A protein was rapidly downregulated when endothelial cells were grown in culture, probably explaining why transcytosis could not be observed in vitro. An attempt to directly demonstrate in vivo transcytosis in mice was inconclusive, using either a cross-reactive anti-ITM2A antibody or in vivo phage panning of an anti-ITM2A phage library.

**Conclusions:**

The present work describes our efforts to explore the potential of ITM2A as a target mediating transcytosis through the BBB, and highlights the multiple challenges linked to the identification of new brain delivery targets. Our data provide evidence that antibodies against ITM2A are internalized in ITM2A-overexpressing HEK293 cells, and that ITM2A is expressed in brain microvessels, but further investigations will be needed to demonstrate that ITM2A is a potential target for brain delivery.

**Supplementary Information:**

The online version contains supplementary material available at 10.1186/s12987-022-00321-3.

## Background

Under normal physiological conditions, endothelial cells form biological barriers that regulate exchanges and maintain a low and selective permeability to fluid and solutes. The endothelial cells lining blood vessels in the brain, unlike those found in peripheral blood vessels, are extremely tightly packed, non-fenestrated and equipped with many efflux systems. These brain endothelial cells are part of the blood brain barrier, which, with its network of tight junctions, efflux pumps and specific metabolic systems, is mostly permeable to very small lipophilic compounds but actively prevents most molecules, in particular large or polar molecules such as biotherapeutics and antibodies, from entering the brain [1, 2]. As a consequence, there are few biologics in drug development in therapeutic areas such as neurology, oncology (e.g., central nervous system (CNS) lymphoma or glioblastoma) or rare brain diseases. For instance, the use of therapeutic antibodies for CNS disorders such as Alzheimer’s, Parkinson’s, and Huntington’s diseases or brain cancers has been very limited so far [3], largely owing to the presence of the BBB. The biologics that are on the market in neurology act peripherally (or else are given intrathecally) [4]. Therefore, strategies to increase brain exposure of biotherapeutics will be key to their success in this field.

So far, the most successful strategy to deliver biotherapeutics to the brain following systemic administration has been to use a ligand or antibody against receptor-mediated transcytosis (sometimes referred to as the ‘Trojan horse’ approach). Several receptors such as insulin [5], transferrin [6], lipoprotein-related proteins [7, 8], low density lipoprotein [9] or Insulin-like Growth Factor 1 [10] receptors have been used in preclinical models and in the clinic for the three first ones. The most advanced results have been reported with the transferrin receptor, with a few clinical candidates in development; one of these, IZCARGO^®^, a fusion of an anti-TfR antibody and iduronate sulfatase has been approved in Japan as an enzyme replacement therapy for the treatment of mucopolysaccharidosis [11, 12]. Several challenges however remain in the field. One of them is linked to the fact that all these receptors are ubiquitously expressed, leading to exposure in other tissues than the brain, which could potentially lead to pleiotropic and adverse effects. Discovering brain-specific mechanisms remains the ultimate unreached goal and is the active focus of current research in this area.

Identifying endothelial cell-specific or enriched membrane proteins is an essential first step towards facilitating drug delivery to specific organs. Two main workflows have been described for the search of new mechanisms of brain delivery. The first strategy relies on transcriptomic and proteomic approaches from either brain microvessels or endothelial cells of human [40], cynomolgus monkey [41], bovine [42], rat [33, 43] or mouse [14, 44–47], including human [34, 48–50] diseased brains. A second approach is based on phenotypic in vitro or in vivo screening of antibodies and peptide libraries displayed in various formats including phage and yeast [51–53]. Only a few attempts have uncovered new brain delivery targets. Proteomics of rodent BECs have led to identifying CD98 heavy chain (a solute carrier) and Basigin (a matrix metalloprotease) along with the previously known LDL Receptor Related Protein 1 (Lrp1) and Insulin receptor (InsR). Phenotypic panning of naive lama single-domain antibody phage display for binding and internalization in primary human BEC versus primary human lung endothelial cells led to the discovery of FC5 and FC44 [52]. It was later shown that FC5 binds to Cdc50A (energy-dependent clathrin endocytosis) [54]. Our approach combines both strategies.

Within the COMPACT IMI consortium (https://www.imi.europa.eu/projects-results/project-factsheets/compact) a collaborative effort aimed at understanding biological differences among tissue barriers and developing drugs to target specific organs, a variety of proteomic, MicroArray and RNA sequencing studies were conducted using human primary endothelial cells from brain, liver and lung [13]. The resulting over 60,000 RNAs were then processed through several filters, downsizing to mRNAs not detected at all in liver and/or lung or with high differential levels in the brain, mRNAs with mostly transmembrane expression, association to the BBB vasculature, expression and selectivity comparable between rodents and humans and a high degree of conservation between orthologs. Finally annotation of human tissue, cell type and membrane localization using several public databases led to a few mRNAs such as those corresponding to basigin [14] and Low-density lipoprotein receptor-related protein 8 (LRP8) [15], which had already been identified as brain transport mechanisms, thus validating the approach. *ITM2A* was among these mRNAs. *ITM2A* had been identified in previous omics studies in rat [33, 34] and pig [35] BECs and reported to be specifically expressed in brain endothelial cells. However, the function of the ITM2A protein remains largely unknown, and it has never been characterized as a transporter in the brain.

ITM2A (alias E25A or BRICD2A) is a 263-amino acid protein with a single transmembrane domain [16]. Its ubiquitous expression is high in thymus, where it was shown to be an activation marker of thymocyte development [17]. ITM2A is mainly believed to be associated with cell differentiation during myogenesis [18, 19], chondrogenesis [20–24] and odontogenesis [25]. The overall homology between mouse and human ITM2A is more than 95% in the extracellular domain [26]. The ITM2A protein has a motopsin-binding Brichos domain within the C-terminal extracellular domain, but the significance of this domain is poorly understood [27]. The Brichos domains appear to bear a chaperone function in different biological situations and has been shown to bind amyloid fibrils [28]. The expression of ITM2A has recently been shown to negatively regulate autophagic flux by inhibiting lysosomal function, through a physical interaction with vacuolar Adenosyl Tri Phosphatase [29].

Transcriptional expression of *Itm2a* in mouse and human brain has been reported to be homogenous in all brain regions (Protein Atlas) and quite specific of endothelial cells versus neurons, microglia, oligodendrocytes or astrocytes as shown in brain RNA-seq databases: *ITM2A* mRNA quantification by RNA-seq from http://www.brainrnaseq.org/ shows high levels in mouse and human brain endothelial cells [30]. In fact, *ITM2A* is commonly referred to as an endothelial cell-specific gene [30]. An analysis of five human and murine cell type-specific transcriptome-wide RNA expression datasets generated within the past several years also identified *ITM2A* as one of the top expressed genes in endothelial cells [31]. A recent single cell RNA-seq analysis of 20 organs in mice also established the *Itm2a* gene as specific for brain endothelial cells [32].

Two precedents point to an association of *Itm2a* with the blood brain barrier. *Itm2a* was identified as microvasculature-specific through the screening of subtractive cDNA libraries from rat brain capillaries versus kidney/liver on one hand [33, 34] and from porcine brain and aortic endothelial cells on the other hand [35]. In addition, the expression of *Itm2a* found in freshly isolated porcine Brain Microvascular Endothelial Cells (BMECs) was shown to be lost when these cells were grown in culture, similarly to some other known BBB markers [35].

ITM2A as an endothelial brain specific transmembrane protein has not been associated in the literature with brain transcytosis or transport. The present report describes the work we undertook to characterize ITM2A and investigate the potential of targeting ITM2A to enhance drug delivery to the CNS.

## Materials and methods

### Animals

Male and Female cynomolgus monkeys (*Macaca* *fascicularis*) aged from 4.8 to 5.9 years were purchased from Le Tamarinier and Noveprim Ltd. (Mahebourg, Mauritius). Six animals were group-housed in aviaries or interconnected mobile cages and two animals were individually housed in interconnected mobile cages. Animals were housed under controlled conditions (20–24 °C, 40–70% humidity, 10–15 renewals per hour of filtered, non-recycled air, 12-h light cycle) with free access to filtered tap water and daily distribution of expanded diet (sodium dodecyl sulfate SDS, France) and fruits or vegetables. The animals from which brain microvessels were harvested had previously been used in pre-clinical studies; they were submitted to a drug washout period of at least 1 month before euthanasia and brain collection.

Male C57BL6/J mice aged from 6 to 8 weeks were purchased from Charles River Laboratories (France).

Pregnant C57BL/6JRj mice were purchased from Janvier Labs (France) between E10 and E12.

Upon arrival, mice were housed individually (for pregnant mice) or grouped (for male mice, 6 animals per cage) in an enriched environment in a pathogen-free facility at a constant temperature of 22 ± 2 °C and humidity (50 ± 10%) on a 12-h light/dark cycle with ad libitum access to food and water.

Male Wistar rats were purchased from Charles River Laboratories (Germany). Upon arrival, rats were housed individually in an enriched environment in a pathogen-free facility at a constant temperature of 22 ± 2 °C and humidity (50 ± 10%) on a 12-h light/dark cycle with ad libitum access to food and water.

### Isolation of brain microvessels from cynomolgus monkey and rodent cortex

Brains from cynomolgus monkeys or rodents were collected immediately following euthanasia and placed in ice-cold Hibernate A medium (ThermoFisher). All subsequent steps were performed at 4 °C and under a biological safety cabinet. Brain cortices were isolated and placed in petri dishes containing ice-cold Hanks’ Balanced Salt solution HBSS. The meninges and the cortical white matter were removed. The collected tissues were transferred into a new sterile container with HBSS, finely minced with a scalpel, and then pelleted by centrifugation (5 min at 600 g, 4 °C). The pellet was resuspended in a collagenase/dispase solution (Roche, Meylan, France, Collagenase 0.1 U/mL; Dispase 0.8 U/mL prepared in (Ca^2+^/Mg^2+^)-free HBSS) containing type I DNAse at 20 U/mL and Tosyl-l-lysyl-chloromethane hydrochloride (TLCK) at 0.147 g/mL, and incubated at 37 °C for 60 min, under vigorous agitation. The digested tissue was carefully homogenized, and centrifuged for 5 min at 600*g*, 4 °C. The resultant pellet was resuspended in HBSS containing 20% Bovine serum albumin (BSA) and centrifuged at for 30 min at 2000*g*, 4 °C. The myelin ring-containing supernatants were discarded, and the vessel-containing pellet was resuspended and re-incubated in the collagenase/dispase solution in presence of DNAse and TLCK for 30 min at 37 °C (except for microvessels from mice). This suspension was re-pelleted by centrifugation 5 min at 600*g*, 4 °C, and the final pellet [named passage 0 Day in vitro 0 (P0D0) fraction from this point onwards] was resuspended in endothelial cell medium (endothelial basal medium EBM-2) supplemented with endothelial cell growth medium micro vascular (EGM-2 MV, Single Quots, Lonza, Basel, Switzerland) containing 3 g/mL puromycin, then seeded in pre-coated (collagen IV 100 g/mL, fibronectin 10 g/mL, Sigma, Saint Quentin Fallavier, France) cell culture flasks, and incubated at 37 °C, 5% CO2 for 7 days. Every 2 days the cell medium was changed; the puromycin concentration was lowered to 2 g/mL after 4 days, and subsequently removed. Following 7 days of expansion at P0D7, Brain Endothelial Cells (BECs) from cortex were further singularized and re-plated de novo for a further 7-day cell expansion (P1D7).

### Stable cell line generation

#### Plasmid constructs

Several human influenza hemagglutinin (HA) human/*ITM2A* cDNA with coding sequence NM_004867 and its variants were synthetically made by GeneART and introduced in a PiggyBac^®^ transposon mammalian expression vector pBH 6450 with a Cytomegalovirus (CMV) promoter. The HA tag was inserted at different positions in the *ITM2A* sequence: at the NH2 or the COOH side or in the Brichos domain; at the NH2 or the COOH side of the Brichos domain or in the middle of this domain, in order to potentially disrupt its function. Plasmids were named: pBH-h*ITM2A* HA NH2, pBH-h*ITM2A* HA COOH, pBH-h*ITM2A* wild type (wt), pBH-h*ITM2A* Brichos HA COOH, pBH-h*ITM2A* Brichos HA NH2, pBH-h*ITM2A*4 Brichos HA mid.

Several Green Fluorescent Protein (GFP) *ITM2A* constructs with coding sequence: NM_004867 for human and NM_008409 for mouse were introduced in pcDNA6.2™C-Emerald (Em)GFP or pcDNA6.2™N-EmGFP expression vectors with CMV promoter and blasticidin resistance. The GFP tag was inserted at different positions in mouse or human *ITM2A* sequences: at NH2 or COOH sides. Plasmids were named: pcDNA6.2™N-EmGFP-h*ITM2A*, pcDNA6.2™N-EmGFP-m*Itm2a*, pcDNA6.2™C-EmGFP-h*ITM2A*, pcDNA6.2™C-EmGFP-m*Itm2a*.

#### Cells and cloning

HEK293/CVCL_0045 cells were obtained from Deutsche Sammlung von Mikroorganismen und Zellkulturen (DSMZ). For thawing: cells were thawed rapidly in a water bath at 37 °C, centrifuged at 900 g for 4 mn, and the pellet was re-suspended in culture medium. Cells were cultured in the following medium: Dulbecco’s Modified Eagle Medium (Gibco 21969; glutamine-free: selection pressure for HA tag or Blasticidin 15 µg/mL: selection pressure for GFP tag); 10% Foetal Calf Serum (Eurobio CVFSVF06 heat inactivated Australian), 100 U/mL Penicillin/Streptomycin Gibco 15140. At confluence, the cells were rinsed with Phosphate-buffered saline (PBS) (− Ca2 +, − Mg2 +), detached by enzymatic treatment (Accutase Sigma A6964 or trypsin) at 37 °C for 3 mn, centrifuged at 900*g* for 4 mn. The pellet was re-suspended in culture medium and diluted 1/10 in fresh medium for seeding in new flasks.

Human Cerebral Microvascular Endothelial Cell (hCMEC)/D3 cells were obtained from Cedarlane. Cells were cultured in the “Cell biologics” medium supplemented with H1168.

Bend3 and Madin-Darby Canine Kidney (MDCK1) cells were donated as a gift from Pr Gumbleton. Cells were cultured in 90% DMEM + GlutaMAX™ with high glucose and sodium pyruvate (Life Technologies—10,569,010), 10% Fetal Bovine Serum (FBS).

HEK293/CVCL_0045 cells were transfected with pcDNA6.2™-*EmGFP* or co-transfected with PiggyBac® transposon expression vectors bearing wild-type or variant *ITM2A* cDNAs and transposase plasmids 6209 (10:1) using Lipofectamine 2000^®^, following the manufacturer’s instructions. Transfections were performed at 50% confluence in 24-well plates with 500 ng of plasmid. For stable transfections, clones were obtained by limit dilution in 96-well plates with blasticidin selection for pcDNA 6.2 or in glutamine-free media corresponding to the glutamine synthase selection marker of pBH 6450. Each individual cell was amplified in a 6-well plate, then *ITM2A* expression was checked first by visual inspection of the cells under a fluorescence microscope to detect the GFP tag, then by Western Blot for all the *ITM2A* constructs.

### Antibody generation

#### Antigen preparation

The human and murine *ITM2A* extra-cellular domain (ECD) gene sequence was synthesized and fused to a human Fc in a mammalian expression vector. After preparation of transfection-grade DNA, a transient transfection of HEK293 cells was performed. After 7 days of culture, the ITM2A-Fc containing culture supernatant was isolated and purified by Protein A affinity chromatography according to standard protocols. After buffer exchange to PBS, the purified antigen was analyzed by UV/VIS spectrometry and SDS-PAGE.

#### Antibody-phage selection

The target protein (ITM2A-human Fc) and negative antigen (Protein N Standard, Siemens, QQIM13) were immobilized onto the wells of an Enzyme-Linked Immunosorbent Assay (ELISA) plate (Corning, 9018) for 1 h at room temperature (RT) (1 µg each). After removal of non-bound antigen, ELISA wells were blocked with a 2% BSA solution for 16 h at 4 °C. After washing of the plates with PBS-T (PBS containing 0.05% Tween 20), the antibody-phage library was added to the immobilized negative antigen and incubated for 1 h at RT to remove Fc specific or polyreactive antibody-phage. Additionally, 5 µg Protein N Standard was added as soluble competitor. Non-bound antibody-phage were recovered and incubated on the immobilized target antigen for 2 h at RT. Non-bound or weakly bound antibody-phage were removed by washing with PBS-T (10x) before antigen-specific phage were recovered by Trypsin (10 µg/mL) elution for 30 min at 37 °C. The antibody-phage were rescued by infection of TG1 cells (OD600 = 0.5) for 30 min at 37 °C. After propagation of the cells for an additional 30 min at 37 °C and 500 rpm, ampicillin (100 µg/mL) and glucose (100 mM) were added to the 2YT culture medium. Bacterial propagation was continued for 1 h at 37 °C and 500 rpm. Then, bacteria were co-infected with M13K07 helper phage, incubated at 30 min at 37 °C followed by another incubation for 30 min, 37 °C and 500 rpm. Double-infected bacteria were centrifuged (4000*g*, 10 min) and the cell pellet was resuspended in fresh 2YT, containing Ampicillin (100 µg/mL) and Kanamycin (50 µg/mL). For the amplification of antibody-phage particles, the incubation was continued for 16 h at 30 °C. Then, the culture was centrifuged (4000*g*, 10 min) and antibody-phage containing supernatant was recovered and used for the next panning cycle. Three panning cycles were performed in total. In each cycle, the number of washing steps was increased (cycle 2: 20 ×, Cycle 3: 30 ×) to increase the stringency of the selection.

#### Antibody screening and sequence analysis

After the third panning cycle, the eluted phage were used to infect XL1 (OD600 = 0.5) for 30 min at 37 °C and streaked out on 2YT agar plates, containing ampicillin (100 µg/mL), glucose (100 mM) and tetracycline (20 µg/mL). Incubation was continued at 37 °C until single colonies were observed. Single clones were isolated and transferred into 96-well plates, containing 2YT, ampicillin (100 µg/mL), glucose (100 mM) and tetracycline (20 µg/mL). The bacteria were cultured for 16 h at 37 °C and 300 rpm. Then, 15 µL of the overnight cultures were used to inoculate new 96-well plates, containing 2YT medium, ampicillin (100 µg/mL), tetracycline (20 µg/mL) and IPTG (50 µM). The bacteria were cultured for 16 h at 30 °C and 300 rpm. The cultures were centrifuged (4000*g*, 10 min) and Single-Chain Variable Fragment (scFv) containing supernatants recovered. The scFv containing supernatants were used for antibody screening. In brief, supernatants were diluted with a 2% BSA solution (in PBS, containing 0.05% Tween20), added to the immobilized antigens in a 384 well ELISA plate (20 ng/well) and incubated for 1 h at RT. After washing, binding of the scFv antibodies to human ITM2A-human Fc, murine ITM2A-human Fc, Protein N Standard or BSA was detected via a Myc-tag using a secondary horseradish Peroxidase (HRP) conjugated antibody. The binding was quantified by TMB reaction and absorbance reading at 450 nm. Target specific antibody clones were isolated, and the DNA sequence of the respective scFv antibody analyzed by Sanger sequencing.

#### Immunoglobulin G (IgG) expression

The VH and VL sequence of selected antibody clones was amplified by PCR and cloned into mammalian IgG expression vectors. After preparation of transfection-grade DNA, a transient transfection of HEK293 cells was performed. After 7 days of culture, the IgG containing culture supernatant was isolated and purified by Protein A affinity chromatography according to standard protocols. After buffer exchange to PBS, purified antibodies were analyzed by UV/VIS spectrometry, SDS-PAGE and flow cytometry analysis for cell binding.

### Transcriptomics

Transcriptome Sequencing (RNAseq) and mRNA Expression Analysis were conducted within COMPACT IMI (Li et al. [13]).

#### RNA samples for library preparation

As described previously by Chaves et al. [36], frozen cell pellets from the cellular P0D0, P0D7 and P1D7 fractions were lysed using QIAzol Lysis Reagent (Qiagen, #79306, Courtaboeuf, France). Total RNA was then isolated from the lysates on a QIAcube instrument (Qiagen) using the Rneasy Mini QIAcube kit (Qiagen, #74116) and following the manufacturer’s instructions. The RNA concentration was determined using the Qubit RNA HS Assay Kit (Invitrogen, #Q32852, Illkirch-Graenstaden, France) and the quality and integrity was assessed on a Bioanalyzer 2100 (Agilent Technology, Les Ulis, France) with an Agilent RNA 6000 nano kit (Agilent Technology, #5067-1511).

#### RNA sequencing (RNAseq)

As described previously by Chaves et al. [36], the RNAseq libraries were prepared with 30 ng of input total RNA using the NEBNext Ultra II Directional RNA Library Prep Kit for Illumina (New England Biolabs, #E-7765S, Évry-Courcouronnes, France) with the NEBNext rRNA Depletion Kit (New England Biolabs, #E6310L) and following the manufacturer’s instructions. The libraries were then paired-end sequenced (75 cycles × 2) on the NovaSeq 6000 instrument (Illumina, Paris, France) using the NovaSeq 6000 SP Reagent Kit (300 cycles; #20027465, Illumina).

#### Reverse transcription quantitative polymerase chain reaction (RTqPCR)

Cells or tissues were lysed with RLT buffer from Qiagen with 1% β-mercapto-ethanol according to the manufacturer’s instructions. mRNA was extracted with Qiagen MiniKit followed by DNase step using the Qiacube robot. Reverse Transcription was achieved from 250 ng of mRNA with High Capacity cDNA Reverse Transcription Kit from AppliedBiosystem (ref. 4,368,813). qPCR was performed from cDNA diluted 1/20 using QuantStudio™ 7 in 384 wells with the TaqMan system standard mode and following the manufacturer’s instructions. Taqman primers and probes were from Thermofisher scientific inventoried assay (ref. 4,331,182): *Itm2a*: Mm00515208_m1; *Actin*: Mm01205647_g1; glyceraldehyde-3-phosphate deshydrogenase (*Gapdh*) Mm99999915_g1; Platelet endothelial cell adhesion molecule (*Pecam*) 1 Mm01242576_m1. Raw data were analyzed with QuantStudio™ Real-Time PCR software.

### Proteomics

#### Immunocytochemistry

Cultured cells were fixed in 4% paraformaldehyde for 15 min, at RT, and subsequently permeabilized and blocked in Odyssey LiCor Blocking Buffer containing 0.2% Triton X-100. Primary antibodies were incubated overnight at 4 °C (anti-ITM2A polyclonal AF4876 and 14407-1-AP, monoclonal non-commercial provider Yumab: Yu093-G04, Yu147-A01, Yu147-E02, Yu147-H07, anti-EmGFP A31852 or A11122, anti-HA 901509), and appropriate secondary antibodies conjugated with Alexa fluorophores (Invitrogen) and Hoechst 33432 (Invitrogen) for nuclei staining were subsequently used for 2 h at RT. Images were acquired on a Perkin Elmer Operetta CLS system.

#### Confocal imaging colocalization

Confocal microscope images were acquired with SP8 LEICA microscope, 40X objectives. For the acquisition ITM2A was directly visualized by EmGFP 488. Organelles were stained with the following primary antibodies: anti Lysosomal-associated membrane protein 1 (LAMP1) ab24170 for lysosome detection, anti-giantin ab37266 for Golgi. Anti-organelle antibodies were detected by staining with appropriate secondary antibody Alexa 633 goat anti mouse A21050 1/1000 or goat anti rabbit A21070 1/1000.

#### Western blot

NHP-derived BEC were lysed using ice-cold radio-immunoprecipitation assay (RIPA) or cell lysis buffer containing protease inhibitor cocktail (ThermoFisher) centrifuged at 15,000*g* for 15 min, and supernatant fractions were collected. Samples were added to SDS and loading buffer then denaturized by heat at 95 °C for 5 min. Denaturized samples were loaded into 4–12% Tris–Glycine SDS-page gels (Invitrogen), and left to migrate for 1 h at 180 V. Samples were then transferred onto polyvinylidene fluoride (PVDF) or nitrocellulose membranes using an iBlot 2 Dry Blotting System (Invitrogen) on the P0 program (20 V for 1 min, 23 V for 4 min, 25 V for 2 min). PVDF membranes were then rinsed with Tris-buffered saline with 0.1% Tween 20 (TBST) and blocked for 1 h in 5% non-fat dry milk in TBST (blocking buffer). Membranes were first probed overnight at 4 °C with primary antibodies in blocking buffer (anti-ITM2A polyclonal AF4876 and 18306-1-AP, EmGFP A11122, anti-HA 901509, anti α-tubulin T9026) and then probed with secondary antibodies diluted in TBST for 1 h at RT (1:10,000 diluted HRP-coupled goat anti-mouse IgG or goat anti-rabbit IgG, GE Healthcare). Following secondary antibody incubation, membranes were rinsed thoroughly with TBST, imaged using a LICOR Odyssey Imager and bands quantified using Multi-Gauge v3.0.

#### Mass spectrometry

Post-mortem human brain samples (occipital cortex, devoid of pathological findings) from three 69 to 79-year-old, male, non-demented, control donors were obtained from external biological resource centers in full accordance with legislation and ethical standards. Microvessels were isolated as described above for monkeys. Tissues or cells were lysed in Preomics 2X buffer then crushed gently with MACS Dissociator (Miltenyi Biotec, program Protein for 1 min). Samples were centrifuged 10 min at 4000*g*, 4 °C, supernatants were collected and diluted in 1X Preomics buffer. Benzonase 1/100 was added and incubated 10 min à 95 °C, 1000 rpm. Samples were centrifuged 20 mn at 13000*g* and supernatants were collected. Proteins were quantified by spectrophotometry using the bicinchoninic acid method and spectraMax i3x. 25 µg of protein were digested on Preomics filter in 50 µL following the manufacturer’s instructions for 3 h at 37 °C; after evaporation, samples were diluted to 0.5 µg/µL in 50 µL LC-Load buffer, vortexed and sonicated. Heavy peptides were added to the sample solution to inject 5 fmol of heavy peptides and 2 µg of proteins. Seven heavy stable isotope labelling (SIL) peptides were spiked in final digest prior to Parallel Reaction Monitoring-Mass Spectrometry analysis on Q-Exactive HF /NanoRSLC 3500. From ITM2A_MOUSE Integral membrane protein 2A Q61500, spiked SIL peptides for detection were: IAFNTPTAVQK, NLVELFGK, EDLVAVEEIR, DLLLGFNK. Spiked SIL peptides used for hTFRC detection were: DSAQNSVIIVDK, LTVSNVLK, SGVGTALLLK, AAAEVAGQFVIK, LTTDFGNAEK.

### Transport assays

#### Internalization assay

HEK293 ITM2A cells were cultured on 96-well plates coated with poly-l-lysine at 50,000 cells/well. After 24 h cells were incubated for 1 h at 4 °C in culture medium with 5 µg/mL of antibody (Anti ITM2A RandDsystem AF4876, Yumab antibodies anti ITM2A Yu147-A01, E02 and H07, Yu093-G04, GFP antibodies A31852 or A11122, anti-HA 901509). Cells were washed with ice cold PBS then incubated for 1 h at 37 °C or at 4 °C for control. Cells were washed twice with ice-cold PBS, then for 2 mn with acidic buffer (glacial acetic acid 1/167 in PBS, pH = 2.5) and finally twice with ice cold PBS. Cells were fixed and stained as described above. Images were acquired with Operetta High Content Screening (PerkinElmer) objective 40X water confocal mode.

#### In vitro transcytosis and permeability measurements brainplotting models

All human samples were provided by Brainplotting [37] (iPEPS, Institut du Cerveau et de la Moelle épinière, Hôpital Universitaire de la Pitié-Salpêtrière, Paris, France) in partnership with Sainte-Anne Hospital, Paris (neurosurgeon Dr. Johan Pallud) and harvested during tumor scheduled resection surgery with written informed consent from the patients (authorization number CODECOH DC-2014-2229). Human brain microvessels were obtained from surgical resections of one patient: a 35-year-old female suffering from a diffuse oligo-astrocytic grade III glioma. Microvessels were isolated from peritumoral or healthy brain tissue using an enzymatic procedure [36] adapted from methods previously published for rats [38, 39]. Briefly, tissue samples were carefully cleaned of meninges and excess blood; then, an enzymatic mix was used to dissociate the tissues and microvessels were isolated by retention on a 10 µmM mesh. Cells were cultured in EBM-2 medium (Lonza, Basel, Switzerland) supplemented with 20% serum and growth factors (Sigma) [38, 39]. After seeding brain capillaries in petri dishes, brain primary microvascular endothelial cells were amplified and seeded (P1D0) on Transwell (Corning) with microporous membranes (pore size: 0.4 mm) in monoculture.

Test (1 µg/mL of internal anti-human/cynomolgus Transferrin Receptor C (TFRC) antibody or anti-ITM2A Yu093-G04) and control antibodies (1 µg/mL mouse IgG, clone MG1-45, BioLegend) were added to the upper chamber on the day adapted to the transport assay defined by Brainplotting as a function of TEER values. Fresh endothelial cell medium with none of these compounds was added to the bottom chamber. Final aliquots from both chambers were taken 240 min following incubation at 37 °C, 5% CO2. Compound levels in the original solutions (t = 0 min), and in the upper and lower chambers (t = 240 min) were determined by ELISA (MESOQuickPlex SQ120, MesoScale Discovery, Rockville, MD, USA). Apparent permeability (Papp) coefficients were calculated using the following formula:

$${\text{Papp }}({\text{cm}}/{\text{min}}) = \left[ {{{\text{V}} \mathord{\left/ {\vphantom {{\text{V}} {\left( {{\text{A}} \times {\text{Cluminal}}} \right)}}} \right. \kern-\nulldelimiterspace} {\left( {{\text{A}} \times {\text{Cluminal}}} \right)}} \times \left( {\text{Cabluminal/t}} \right)} \right]$$

where V = volume of cell medium in the bottom chamber (mL), A = surface area of the insert (cm^2^), Cluminal = compound concentration loaded in the upper chamber (µM), Cabluminal = compound concentration measured in the bottom chamber (µM); t = time of the assay (min).

#### ELISA immunoassay (in vitro and in vivo studies)

Standard 96-well sector plates (Meso Scale Discovery) were coated with 0.5 μg/mL of Fab’2 anti-Human (709–006-098 Jackson Immuno Research Labs) or anti-mouse (M0284 Sigma-Aldrich) IgG in PBS and then incubated for 1 h under agitation at RT. After incubation, plates were washed three times with PBS-Tween 0.05% (Calbiochem, 524653) and blocked for 1 h at RT with 0.1% BSA solution (A7030, Sigma). After blocking the plates, samples collected in the transport assay and standards were incubated on plates for 2 h at RT. After incubation, plates were washed three times with PBS-Tween 0.05%, and bound antibody was detected with SULFO-TAG conjugated goat anti-mouse antibody (R32AC-1, Meso Scale Discovery) or goat anti-human antibody (R32AJ-1, Meso Scale Discovery) using Tripropylamine containing read buffer (R92TC-2, Meso Scale Discovery). Concentrations were determined from the standard curve using a four-parameter non-linear regression program (Discovery Workbench version 4.0 software).

### In vivo experiments

#### Mouse brain collection

Each mouse was anaesthetized in an isoflurane gas chamber then perfused through the heart with Li-heparinate solution at a final concentration of 20 U/mL in sterile PBS, using 48 mL of perfusion solution delivered at 8 mL/mn. Brain samples were collected. Each mouse was decapitated immediately after perfusion. The perfused brain was removed, cerebellum and brainstem were separated and eliminated. Brain cortices were washed in ice-cold PBS, collected in pre-weighed Precellys tubes and stored immediately at − 80 °C or on dry ice until use. Then, the pre-weighed hemispheres were thawed and homogenized in 5 vol. (v/w) of brain lysis buffer (1% NP-40 in PBS containing complete mini ethylene diamine tetra-acetic acid—free protease inhibitor cocktail tablets, Pierce) using a bead homogenizer. Homogenized brain samples were then gently agitated at 4 °C for 1 h before centrifugation at 20,000*g* for 20 min.

#### Pharmacokinetic study in vivo in mouse

Five–ten mg/kg (35–70 nmol/kg) in a single dose of anti-ITM2A (clone Yu093-G04) or control [anti-trinitrophenyl (TNP), batch VA2-17-419-1, internal production] antibodies were administered by intravenous caudal injection into C57Bl/6 male mice weighing 20-25 g (n = 3/condition). 5 h post-injection, plasma and saline-perfused brains were collected and antibody concentrations were determined by ELISA as described above.

#### In vivo panning in mice

Yumab provided an amplified phage library to Sanofi. First, a naïve human antibody phage library was enriched for antigen-specific antibodies against ITM2A proteins. The antibody phage output was amplified and purified by poly-ethylene glycol (PEG)/NaCl purification. Purified antibody phages were directly used for the in vivo panning: 10^11^ antibody phage particles (~ 10 µl) were mixed from each panning output. The antibody phage mix was injected into the mouse. Brains were isolated after 1 h and 24 h (2 × each). Brains were collected as described above.

Antibody phages in the homogenate were used for infection of *E.* *coli*. Bacteria were selected and used for production of monoclonal scFv antibodies. About 800 clones were picked and screened by scFv in ELISA.

### Statistical analysis

The statistical significance of differences between groups was analyzed using GraphPad Prism v9.0.0 software (GraphPad Software, San Diego, CA, USA) and Ordinary one-way Analysis of Variance (ANOVA) (non-parametric or mixed) with Dunnett’s multiple comparisons test, Ordinary two-way ANOVA (mixed) with Sidak’s, Kruskal Wallis or Mann–Whitney multiple comparison test or unpaired t test (non-parametric). The application of these statistical methods to specific experiments is noted in the figure legends.

## Results

**Fig. 1:**
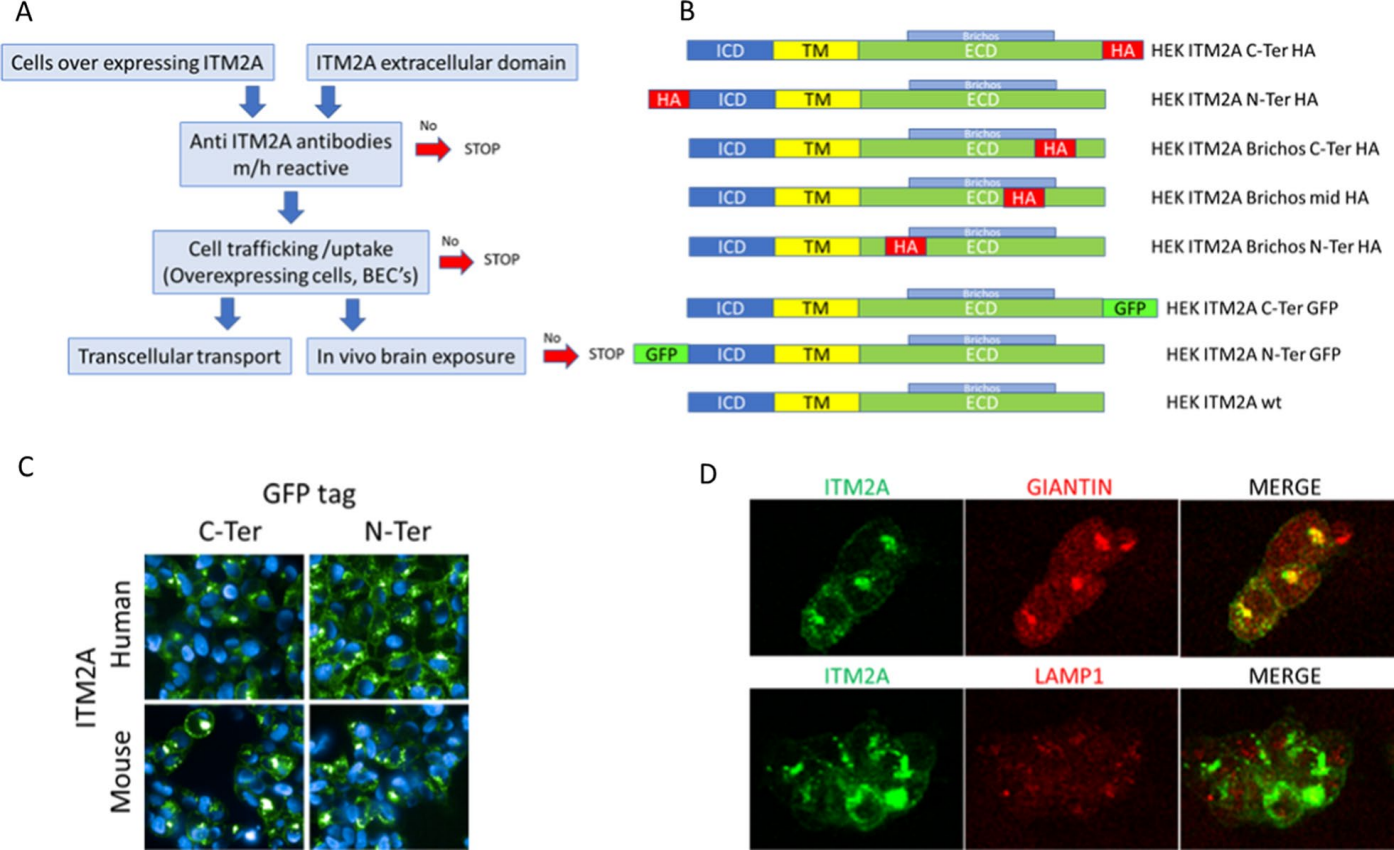
Validation of ITM2A as a potential brain delivery target and stable cell line generation. **A** Flowchart, go steps are highlighted with blue arrow, no-go steps are highlighted with red arrow. **B** Human or mouse tagged ITM2A plasmid design. Each construct has intracellular domain (ICD) in blue, transmembrane domain (TM) in yellow and extracellular domain (ECD) in green with Brichos domain; HA tag localization in red, GFP tag in fluo green. **C** Human or mouse GFP tagged ITM2A expression in HEK293 cells; HEK293 cells are stably recombined with human or mouse ITM2A with a GFP tag at different positions; GFP-tagged ITM2A is visualized in green, and nuclei are stained in blue by Hoechst. **D** Highlighting the ITM2A colocalization in HEK293 human ITM2A N-Ter GFP cell compartments. Confocal images of N-Ter GFP ITM2A in green and anti-Giantin antibody (ab37266) in red or anti-LAMP1 antibody (ab24170) in red. Yellow color indicates colocalization in the same focal plan, red and green colors indicate no colocalization in the same focal plan

Our strategy is outlined in Fig. 1A: the first objective was to produce tools to study the mechanisms, cells overexpressing ITM2A and the extracellular domain of the protein to enable producing anti-ITM2A antibodies with affinity for both human and mouse ITM2A. With these antibodies, experiments measuring uptake, internalization, and trafficking in either the above overexpressing cells or in BECs provide the first filter. Subsequent evidence of transcytosis and in vivo brain pharmacokinetics would lead to final validation of ITM2A as a potential brain delivery target (Fig. 1A).

### Generating cell lines overexpressing ITM2A

In the absence of available monoclonal antibodies, we devised a strategy that could bring clues on the potential of this protein to carry biologics into the brain using specific well-characterized cargos. To this end, we engineered several constructs of human or murine *ITM2A*. One plasmid, named wt, was designed with no tag, the others bearing either an HA-tag (i.e., amino acids 98–106 of human influenza hemagglutinin glycoprotein) or a GFP-tag at different positions, namely on the C-Terminal (C-Ter) extracellular domain or on the N-Terminal (N-Ter) intracellular domain of ITM2A; in addition, the HA tags were positioned at three distinct locations of the Brichos domain within the extracellular portion (Table 1) to bring additional insight on the potential position involved in internalization. We selected adherent HEK293 cells since they are well suited for immunofluorescence analyses and produced 10 clonal cell lines by recombination with the various tagged or untagged mouse or human plasmids displayed in Fig. 1B and Table 1. Clones were obtained after limit dilution and selection by either fluorescence and Western Blot for GFP tag or by Western Blot for HA tag and wt *ITM2A* (Additional file 1).

**Table 1:** Engineered HEK293 cells overexpressing various tagged or untagged human or mouse *ITM2A*

Mouse *itm2a*		Human*ITM2A*	
GFP tag	HA tag	GFP tag	No tag
N-Ter C-Ter	N-Ter C-Ter Brichos 1 C-Ter Brichos 2 N-Ter Brichos 3 Mid	N-Ter C-Ter	wt

### Fluorescence visualization of ITM2A

After cell line validation for the ITM2A construct protein expression by Western Blot (WB), cellular localization of both human and mouse ITM2A was visualized by fluorescence microscopy in cell lines using GFP fluorescence (Fig. 1C). GFP fluorescence was detected in all N-Ter and C-Ter constructs in HEK293 cells overexpressing either human or mouse ITM2A.

### Cellular localization of ITM2A

Aside from membrane localization, ITM2A was found to be localized in the Golgi system of the overexpressing HEK293 cells, as shown by co-localization with Giantin (Fig. 1D). No ITM2A could be seen in the lysosomes as shown by LAMP1 co-labeling (Fig. 1D).

### Cell uptake of antibodies

With these cell lines in hand, we went on to evaluate their capacity to uptake antibodies after binding with the protein, using several approaches.

**Fig. 2:**
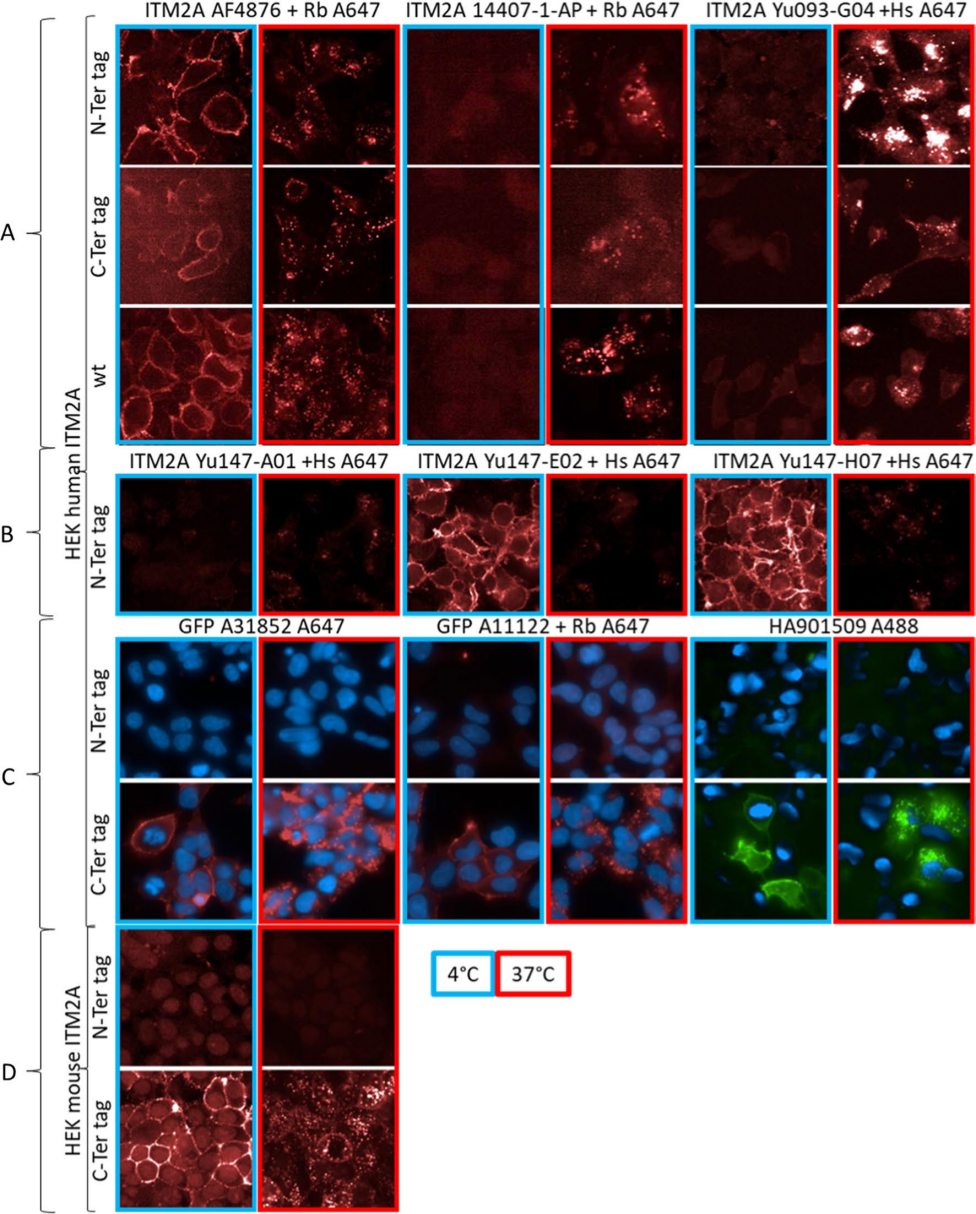
ITM2A uptake and internalization of several antibodies (anti-ITM2A, anti-GFP or anti-HA) on HEK293 human and mouse. **A**, **B** internalization of anti ITM2A revealed with anti-species Alexa 647 in red on HEK293 hITM2A wt, hITM2A GFP N-Ter and hITM2A GFP C-Ter. **C** internalization of anti-tag antibodies: anti-GFP A31852 Alexa 647 in red, anti-GFP A11122 revealed with anti-Rabbit Alexa 647 in red and anti-HA 901509 Alexa 488 in green on HEK293 hITM2A wt, hITM2A tag N-Ter and hITM2A tag C-Ter. **D** internalization of anti GFP A31852 revealed with anti-Rabbit Alexa 647 in red on HEK293 mouse ITM2A

First, we performed immunofluorescence studies on HEK293 cells overexpressing human ITM2A with two commercially available polyclonal antibodies against extracellular epitopes of human ITM2A: AF4876 and 14407-1-AP (Fig. 2A). The first one displayed membrane labeling at 4 °C while the second one could not be detected at 4 °C, possibly due to a lower affinity or different behavior after acid washing (performed before analysis). Both antibodies readily internalized at 37 °C leading to punctate labelling, demonstrating active uptake. The weaker signal observed with AF4876 on the C-Ter GFP cell line could result from steric hindrance of the GFP tag for antibody binding. The same results were obtained using fluorescent anti-GFP antibodies (A31852), or after detection of an unlabeled anti-EmGFP antibody (A11122) with a secondary labeled Alexa 647 anti-Fc rabbit antibody (Fig. 2C). In both cases, very clear membrane labeling could be observed at 4 °C followed by internalization at 37 °C with the HEK293 cells overexpressing human and murine C-Ter GFP ITM2A, but not N-Ter GFP ITM2A, confirming the need for the GFP tag to be extracellular for recognition.

We generated analogous results in HEK293 cells with anti HA antibodies (Fig. 2A), where clear internalization could be observed when the label was in C-Ter, leading to punctate labelling, confirming that ITM2A was functional and able to internalize cargos such as antibodies into cells.

Similar results were obtained with anti GFP antibodies and mouse ITM2A GFP constructs in HEK293 cells overexpressing mouse ITM2A with C-Ter and N-Ter GFP demonstrating functional internalization of mouse ITM2A (Fig. 2D).

### Production of monoclonal ITM2A antibodies

A campaign was launched in collaboration with Yumab and the COMPACT IMI consortium to generate mouse/human cross-reactive monoclonal antibodies. The genes of the murine and human *ITM2A* ECD were synthesized and fused to the human IgG1 Fc part on a mammalian antigen expression vector. After transient transfection of HEK293 cells, the ITM2A-Fc fusion proteins were expressed, secreted by the cells into the culture medium and purified. A total of four different antibody selections were performed using two antibody libraries consisting of human kappa or lambda antibodies, comprising together a diversity of more than 10^10^ different antibody sequences.

From 2304 clones tested for binding activity on the murine and human ITM2A, 220 cross-reactive and 15 mouse-specific hits were identified. 19 cross-reactive antibodies with unique sequences were selected, produced and purified as fully human IgG in HEK293 cells. Testing these antibodies on human ITM2A overexpressing HEK293 cells revealed a low correlation between binding to the recombinant protein and cell binding. Four IgG antibodies did exhibit potent cell binding. These antibodies could demonstrate binding and internalization similar to those shown previously. For instance, when HEK293 cells overexpressing wt, C-Ter or N-Ter GFP-ITM2A were exposed to Yu093-G04, internalization occurred readily at 37 °C (Fig. 2A). Similarly Yumab Yu147-A01, Yu147-E02 et Yu147-H07 and the control AF4876 displayed binding at 4 °C to HEK293 cells. Upon washing and warming to 37 °C they were internalized, as evidenced by a punctiform labeling inside cells (Fig. 2B).

### ITM2A expression in BECs

With antibodies demonstrating binding and uptake in hand, our next step was to evaluate their capacity to perform transcytosis. Before testing our ITM2A antibodies in models of transcytosis, we checked whether we could find ITM2A expression in our BECs.

**Fig. 3:**
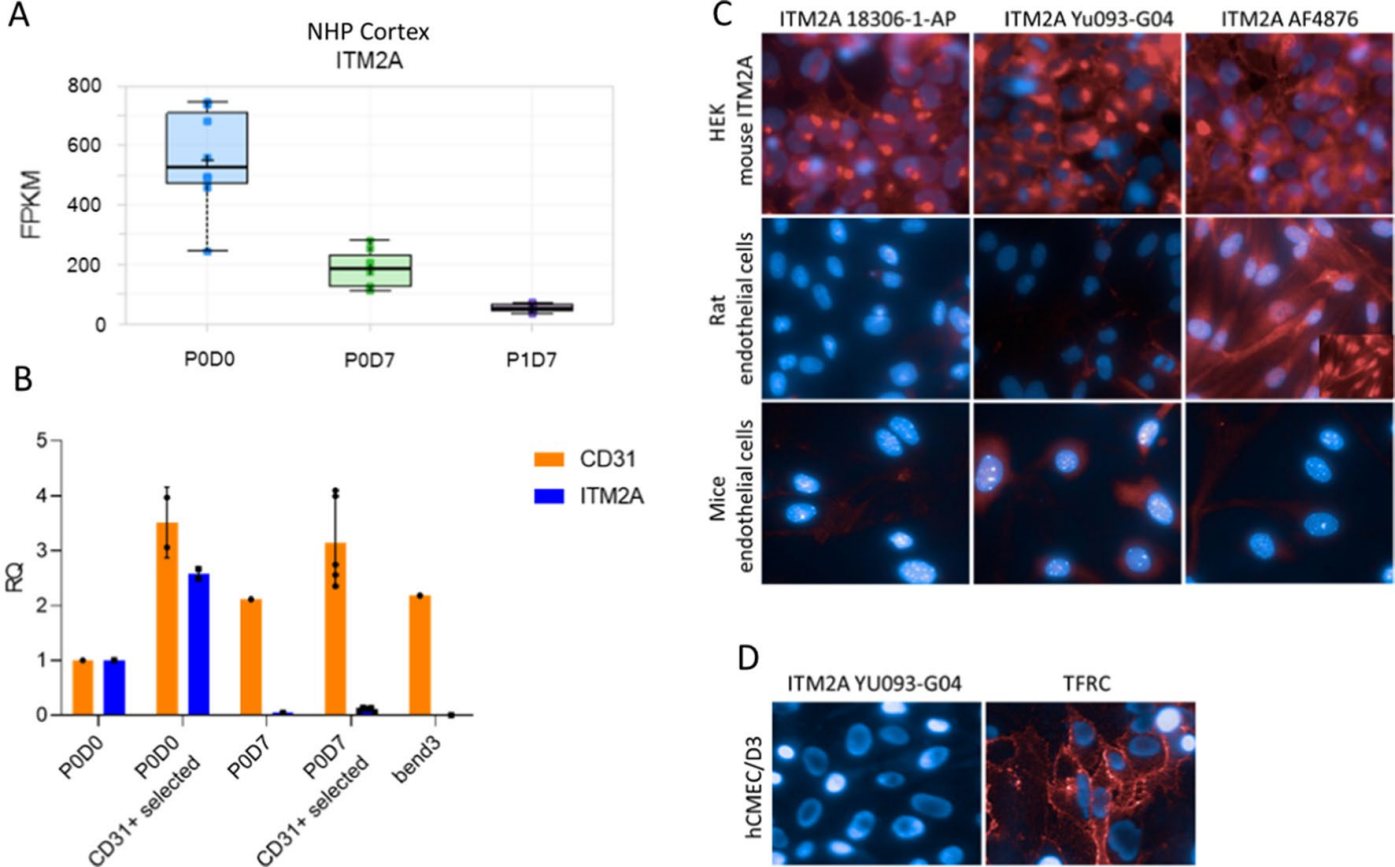
ITM2A mRNA and protein detection in endothelial cells from different species. **A** ITM2A mRNA detection in monkey endothelial cells from cortex analyzed with RNA-seq at different times of culture. P0D0 (blue) = fresh microvessels, P0D7 (green) = endothelial cells after 1 week in culture, P1D7 (magenta) = endothelial cells after 1 thawing then 1 week in culture. **B** Differences in the mRNA levels of Itm2a (orange) and CD31 (blue) in mouse endothelial bEnd.3 cells. Microvessels named P0D0, primary cells selected by puromycin named P0D7, primary cells selected by CD31 + named P0D0 CD31 + selected and P0D7 CD31 + selected. Actin was used as a housekeeping gene. **C** Immunofluorescence staining of mouse ITM2A protein in rodent endothelial cells with three ITM2A antibodies. **D** Immunofluorescence staining of hCMEC/D3 with TFRC and ITM2A antibodies. Labelling is visualized with secondary anti-species Alexa 647 antibodies in red. Nuclei are labelled with Hoechst in blue

First, we assessed *ITM2A* mRNA levels in brain endothelial primary cells and cell lines. mRNA levels were rapidly lost when non-human primate brain endothelial cells were grown in culture (Fig. 3A, cortex shown). Similar downregulation after culture was also observed in mouse primary BECs (Fig. 3B). In comparison, cluster of differentiation 31 (CD31), a specific marker of endothelial cells, retained high expression throughout the culture. This downregulation most certainly also explains why no expression of mouse *Itm2a* could be found in bEnd.3 cell lines (Fig. 3B).

**Fig. 4:**
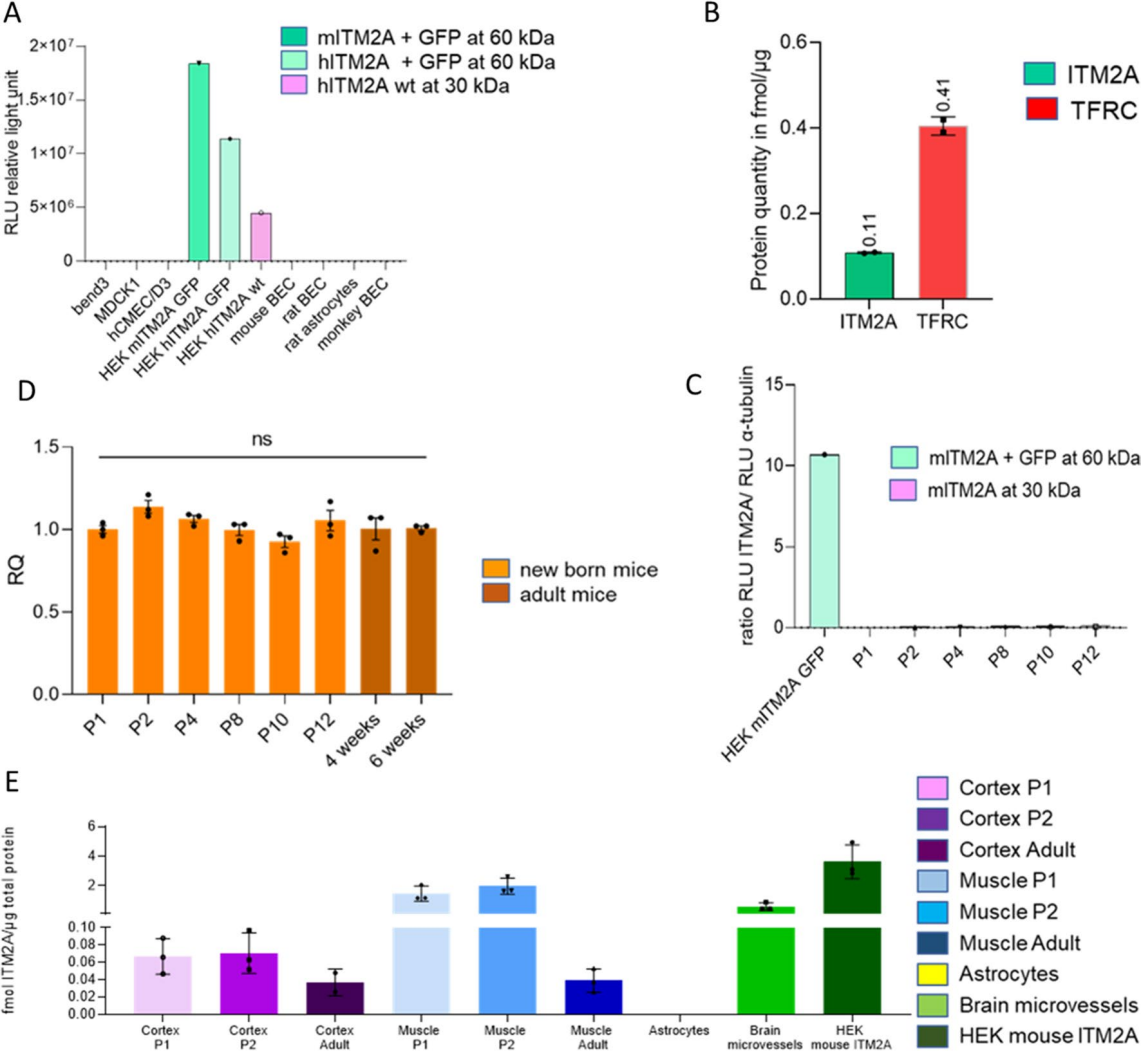
ITM2A RNA and protein levels. **A** Relative quantification of ITM2A protein expression in different cells by Western Blot. Green: protein at 60 kDa (ITM2A + GFP), pink: protein at 30 kDa (ITM2A). Western Blot membranes are in Additional file 2. **B** Quantification of ITM2A in samples of human brain microvessels by LC/MS. ITM2A in green and TFRC in red are expressed as mean ± S.D. (4 quantified peptides for ITM2A and 5 quantified peptides for hTFRC). **C** Relative quantification of ITM2A protein expression in newborn mice by Western Blot. HEK293 mouse ITM2A GFP was used as control. Green: protein at 60 kDa (ITM2A + GFP), pink: protein at 30 kDa (ITM2A). Western Blot membranes in Additional file 3. **D** itm2a mRNA relative quantity (RQ) in new-born and adult mouse brain homogenates. Housekeeping gene was gapdh. Results are expressed as mean ± S.D. (n = 1 experiment, performed in triplicates). p values were obtained by Ordinary One-way ANOVA with Dunnett’s multiple comparisons test vs P1. **E** Quantification of ITM2A in samples of mouse cells and tissues by LC/MS. Cortex in purple, muscles in blue and cells in green. Results are expressed as mean ± SD. (3 quantified peptides)

Likewise, we could not detect endogenous ITM2A protein in primary endothelial cells from brain cortex of mouse or rats by immunolabelling compared to mouse ITM2A overexpressing HEK293 cells which displayed nice immunolabeling with anti-mouse ITM2A antibodies (AF4876, 18306-1-AP and Yumab Yu093-G04). (Fig. 3C). No signal or a non-specific signal was detected in primary cells, in contrast to HEK293 cells overexpressing ITM2A. Similarly, we could not detect endogenous ITM2A protein in hCMEC/D3 by immunolabelling compared to TFRC, probably due to the downregulation of *ITM2A* in culture (Fig. 3D). As immunolabelling showed no signal for ITM2A, we checked the level of ITM2A by WB using commercially available polyclonal antibody AF4876 and as a control we evaluated the mouse ITM2A-GFP fusion in HEK293 cells using an anti GFP antibody. The protein was well detected at the expected molecular weight (MW) (30 kDa for ITM2A + 27 kDa for GFP) in the HEK293 ITM2A-overexpressing cells but could not be detected in mouse, rat or monkey primary endothelial cells or astrocytes, nor could it be detected in other cell lines (bEnd.3, MDCK1, hCMEC/D3) (Fig. 4A).

We checked *itm2a* mRNA expression in mice of different ages but could not see any marked difference. Detectable expression [*itm2a* Cycle Threshold (Ct) = 25 vs *gapdh* Ct = 21] was present throughout post birth (P1) to six weeks of age in the cortex of mice (Fig. 4D). However, when these brain fractions were analyzed by WB, no band at 30 kDa could be identified (Fig. 4C).

### Proteomic studies to quantify ITM2A

To clarify whether ITM2A protein was or not present in the mouse, proteomic studies were conducted in mouse cortex and muscle of newborn (P1 and P2) and adult mice, along with freshly prepared astrocytes and brain microvessels in comparison with HEK293 cells overexpressing mouse ITM2A. By Liquid Chromatography/ Mass Spectrometry (LC/MS), six endogenous peptides belonging to C-Ter or N-Ter ITM2A were used for detection and quantification. The results are shown in Fig. 4E. While ITM2A was well detected in newborn muscle samples and in brain microvessels, less ITM2A was detected in both newborn and adult cortex samples and in adult muscle samples and no ITM2A was detected in astrocytes. After quantification of ITM2A in each sample, protein intensities were calculated by averaging peptide intensity values (Fig. 4E). Newborn cortex and muscle, brain microvessels and HEK293 cells overexpressing mITM2A averaged 0.1; 1.7; 0.6 and 3.6 fmol of ITM2A per µg of total protein content, respectively. From these experiments, it was concluded that ITM2A could be quantified in mouse brain and muscle albeit to a much lower extent in adult muscle and in cortex from newborn and adult. The protein was higher in brain microvessels freshly isolated from adult wild-type mice than in cortex homogenates, pointing to endothelial cell enrichment. Levels of TFRC in mouse brain microvessels were in the same range (0.8 fmol/µg). These results suggest that WB conditions might not have been sensitive enough to detect such levels of ITM2A. This was verified by diluting lysates of HEK293 cells overexpressing mouse ITM2A. From the above-determined levels of 3.6 fmol ITM2A/µg of protein we determined that our limit of detection using Western blotting (Additional file 4) was 7.2 fmol of ITM2A, over ten-fold the levels found in brain microvessels. Finally, ITM2A was quantified in human microvessels and found to be four times lower than TFRC (ITM2A 0.11 fmol/µg of total protein vs TFRC 0.4 fmol/µg of total protein, which could suggest lower transcytosis efficacy (Fig. 4B).

### In vitro transcytosis in human primary BBB model

As the ITM2A protein could be quantified, we decided to study one of our anti-ITM2A antibodies in a transcytosis model. Because of the strong ITM2A downregulation in cultured cells, cell line derived models were excluded. Even primary models which require a minimal culture to access enough endothelial cells were ruled out as we showed that in our NHP primary model ITM2A was already strongly downregulated. Preparation of human primary endothelial cells suitable for transcytosis was difficult owing to the challenge of obtaining very fresh post-mortem human brains. We decided to turn to a human primary BEC model prepared from freshly resected brain tissue from glioblastoma or epilepsy surgeries provided by Brainplotting.

**Fig. 5:**
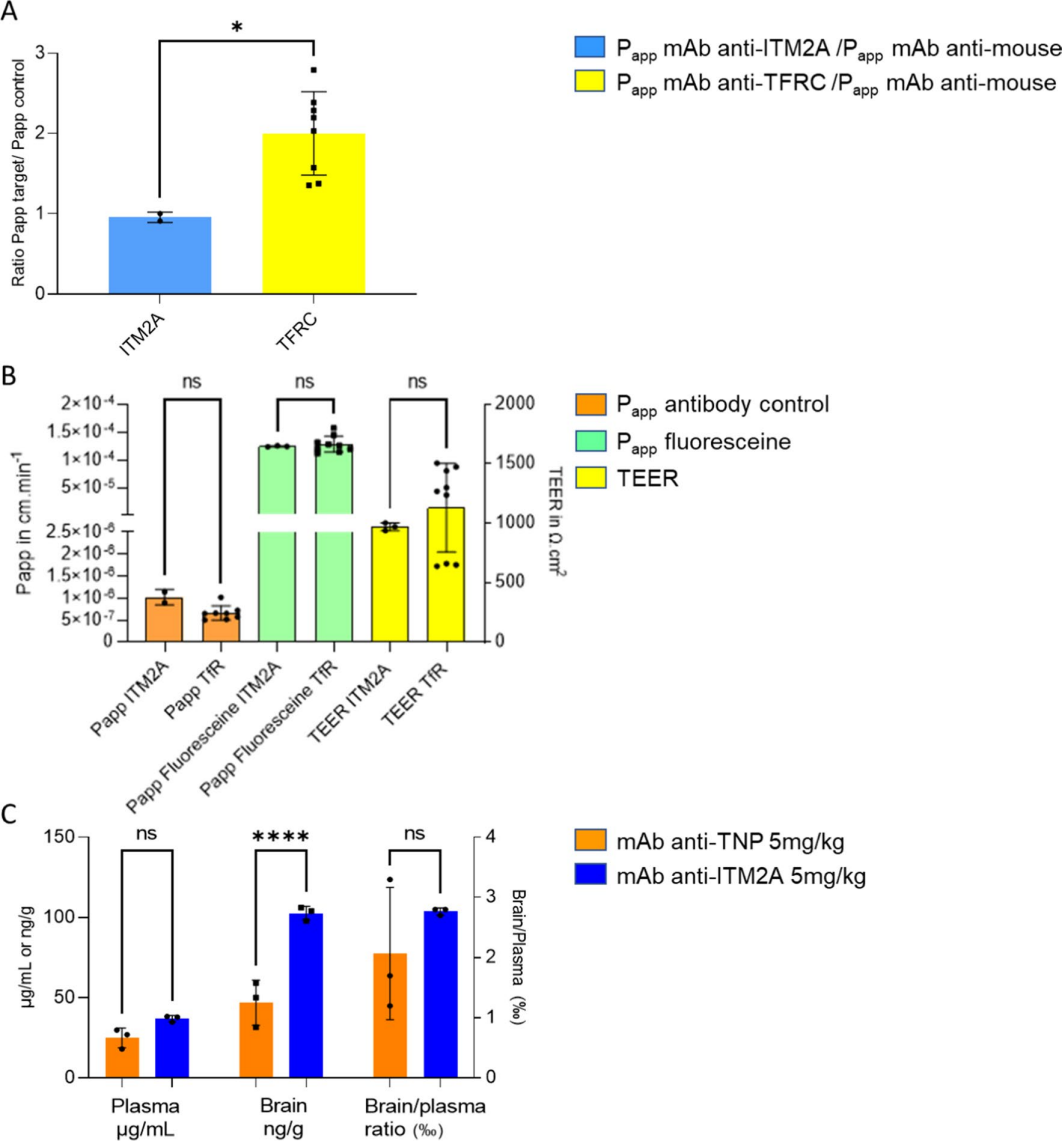
ITM2A transport assays. **A** In vitro human brain transcytosis model from BrainPlotting. P_app_ antibody anti- ITM2A in blue and P_app_ antibody anti-TFRC in yellow normalized to P_app_ Control mouse IgG. Results are expressed in P_app_ ratio (target/control) means ± Standard Deviation p values were obtained by Mann–Whitney test *p = 0.0444. **B** In vitro human brain transcytosis controls were Papp control antibody, Papp Fluoresceine and TEER measurements in each Transwell. Results are expressed in means ± Standard Deviation p values were obtained by Mann–Whitney test NS: p > 0.9999. **C** In vivo mouse brain exposure of an ITM2A monoclonal antibody. 5 mg/kg iv injection of Yu093-G04 and anti-TNP to C57Bl6 mice; samples were collected 5 h after injection. Results are expressed as mean ± S.D. (1 experiment, n = 3 animals). p values were obtained by Two-way ANOVA with Sidak’s multiple comparisons test ns: no statistical significance = p > 0.05, ****p < 0.0001

The anti ITM2A Yu093-G04 antibody was evaluated in the model. As illustrated in Fig. 5A, the antibody did not perform better than a control antibody. In this model, an anti-TFRC receptor antibody systematically performed better than the control antibody. As shown in Fig. 5B, controls such as P_app_ control antibody, P_app_ fluoresceine and TEER measurement were not significatively different in Transwell incubated with anti-ITM2A antibody Yu093-G04 or with anti-TFRC antibody.

### In vivo mouse brain exposure of ITM2A antibodies

To finally conclude on the potential of this protein to enhance brain exposure of antibodies, two in vivo mouse experiments were conducted.

A single administration study in mice of the anti ITM2A monoclonal antibody (mAb) Yu093-G04 versus a control anti-TNP mAb was performed and levels of the mAbs in brain and plasma after 5 h were determined by ELISA. There was a weak (≥ two-fold) increase in brain exposure of the anti-ITM2A mAb compared to the anti-TNP (control) antibody (Fig. 5C).

In a second experiment, a naive human antibody-phage library was enriched for antigen-specific antibodies against ITM2A. The antibody-phage output was amplified and purified by PEG/N antibody purification. The purified phage display library of more than 6 million phages (panning campaign PC084, Strategy S1-1-10, panning rounds 3, diversity 6.3.10^6^, titer ~ 1.10^13^ cfu/mL) anti ITM2A ScFv’s was used for in vivo panning and injected in mice. Brains were isolated after 1 h and 24 h and the brain homogenates were used to perform infection of *E.* *coli*. Picking of ~ 800 clones, screening of scFv supernatant in ELISA and on cells did not allow to identify any ITM2A phages in the brain.

## Discussion

To investigate a putative transport mechanism for ITM2A along with potential for delivering drugs to the brain, we first developed cells overexpressing the protein with the aim to determine its localization and to assess binding and uptake of anti-ITM2A antibodies. At the start of this effort, we did not have access to monoclonal anti-ITM2A antibodies. Several anti-ITM2A antibodies were reported [17, 29] or commercially available but most were polyclonal. We reasoned that engineering the protein with GFP and HA tags at the C-Ter (extracellular domain) position of the protein, could serve the double purpose of visualizing its cellular localization using GFP fluorescence and allow the study of binding and uptake with antibodies against these tags. This strategy of tagging a protein to circumvent the absence of monoclonal antibodies has actually been reported for ITM2A and has helped decipher its role in the hedgehog signalling pathway [40]. Tags at the N-Ter were also engineered as controls along with several positions within the extracellular Brichos domain, toward bringing information on the precise site involved in endocytosis. Exposure of the cells to anti-GFP antibodies first allowed to confirm ITM2A’s plasma membrane localization. Cellular localization of ITM2A has been looked at in a few cell systems and shown at the plasma membrane, in large cytoplasmic vesicles, possibly endosomes, and in the Golgi system [17]. In particular, cytosolic localization has been reported in HEK293 cells overexpressing ITM2A where the protein could be found colocalized with LAMP1 in lysosomes [29]. We confirmed the presence of ITM2A in the Golgi system but not in lysosomes in our colocalization experiments using GFP tags. In addition to antibody binding, HEK293 cells demonstrated nice uptake of anti GFP or HA antibodies respectively. Both binding and uptake were specific of cells overexpressing ITM2A proteins bearing extracellular tags. The cells overexpressing ITM2A proteins bearing intracellular tags did not lead to binding or uptake with these antibodies, confirming that this uptake was specifically linked to extracellular binding to ITM2A. Internalization of receptors genetically engineered with extracellular tags such as HA, cMyc, EGFP, has been documented in the literature with some G-coupled receptors [41], Transforming Growth Factor β [42] or erythropoietin [43] receptors. However, this uptake is far from systematic, and many antibodies do not internalize upon binding their antigen receptors as shown for instance by Jacobsen et al., with an anti-Myc antibody and myc-engineered GPR6 and β2-adrenergic receptors [44]. The specific uptake of these antibodies upon binding to the extracellular part of the ITM2A protein was interpreted as a positive signal and gave us the second go for our validation flowchart. Monoclonal anti ITM2A antibodies were later designed and generated using the extracellular domain of ITM2A as antigen and the resulting antibodies confirmed the uptake seen previously.

Our next objective was to demonstrate that this uptake could lead to transcytosis in polarized endothelial cells. For this we needed a human or rodent transcytosis model overexpressing the protein, since our anti ITM2A antibodies are cross-reactive. From the COMPACT IMI consortium studies, it had been shown that the protein could no longer be found after one cell passage. We show here that *ITM2A* expression is strongly downregulated in cultures of either non-human primate [36] or mouse primary BEC’s (Fig. 3A, B) as is the case for several BBB genes after cell line establishment or culture [45, 46] and has already been shown specifically with *ITM2A* [35].

Most expression studies reported in the literature about ITM2A are transcript-based. In humans, a substantial amount of the *ITM2A* transcript can be found in the brain as detailed in databases such as Gtexportal, Stanford, GenCard or the Open Targets Platform. In addition, according to Zhang et al. in humans, the amount of *ITM2A* mRNA in endothelial cells was evaluated at 150 Fragments Per Kilobase Million (FPKM) versus 23 FPKM for *TFRC,* a well-known transcytotic receptor, while in total brain the two proteins accounted for 2 FPKM. In mice, the amount of *ITM2A* mRNA was evaluated at 2000 FPKM in endothelial cells against 800 FPKM for *TFRC* and respectively 80 and 60 FPKM in the total brain for the two proteins [30].

Our own RNA-seq study in non-human primate brain microvessels had shown intermediate expression of *ITM2A* in cortex, hippocampus and septum (FPKM 9–16) and very low expression in liver [36]. By comparison in the same study, *TFRC* had shown a high expression (FPKM 75–125) in brain structures [36]. Mitsui [27] reported that the ITM2A protein was strongly detected in the lysates of mouse cerebral cortex between P0 and P10, and gradually decreased towards adulthood. Our own experiments in mice from P0 to 6 weeks of age showed that their *Itm2a* mRNA content remains constant throughout age. However, the ITM2A protein could not be detected by WB in any of the samples, nor could it be detected in mouse, rat or monkey primary endothelial cells or astrocytes or in other cell lines (bEnd.3, MDCK1, hCMEC/D3) as opposed to the HEK293 cells overexpressing ITM2A where a strong band could be seen (Fig. 4A). The theoretical MW of ITM2A is of ~ 30 kDa (Gencards or Proteintech) and it was reported that post translational modifications lead to an apparent MW of 43 and 45 kDa, probably resulting from N-glycosylation at amino acid position 166 [17]. However, no protein could be detected around this MW either. Fluorescence gave the same results with a strong signal for the HEK293 cells and no signal for endothelial rat or mouse cells or hCMEC/D3 cells, confirming results from Masuda et al. [47].

Using more sensitive proteomics, we were able to measure the levels of ITM2A in mouse brain microvessels. These levels have been confirmed to be under the limit of detection of our WB conditions. Nevertheless, as these levels were in the same range as the ones found for TFRC in the same study, we considered that it was worth conducting a transcytosis experiment.

One of our monoclonal anti-ITM2A antibodies was therefore evaluated in a model based on primary cultures of human BMEC from BrainPlotting [37]. These cells are prepared from fresh brains derived from surgical resections and are used after a very short time in culture, which could reduce the changes in phenotype seen when these cells are cultured for longer durations. In this model, a TFRC antibody reproducibly showed enhanced apparent permeability versus a control antibody. However, when the ITM2A antibody was evaluated in the same conditions, no difference in permeability versus the control was observed (Papp ITM2A 0.97.10-6 versus control 1.02.10^–6^ cm.min^− 1^). As we had no information regarding the status of ITM2A levels in this model, we could not clearly conclude on this experiment.

The area of predictivity of in vitro blood brain barrier models is still a matter of intense research and debate. Even if brain exposures of some antibodies have been linked to their apparent permeabilities in in vitro transcytosis models [48, 49], this was shown in cases where in vitro and in vivo experiments were performed in the same species as described by Stanimirovic et al. in rat [48]. In vivo brain exposure, distribution and pharmacokinetics are dependent on a series of dynamic processes, linked also to target engagement, localization and cellular trafficking. All these would be difficult to recapitulate in an in vitro model, and even more so for species-to-species predictions where additional parameters such as anatomy, capillary bed density, molecular composition, as well as the density of specific BBB transporters [48] need to be considered.

To conclude about the potential of ITM2A to transport antibodies to the brain, we used one of our specific anti ITM2A antibodies directly in vivo to determine its brain exposure in mice. When injected at 5 mg/Kg Yu093-G04 could be measured in brain parenchyma 5 h after injection with a two-fold higher level than a control antibody. The brain/plasma ratios were not very different between ITM2A and control antibodies (both around 0.3%). This ratio was in the range of what is described in the literature regarding brain/plasma ratio for antibodies with no modification to enhance brain delivery: 0.1% in the rat [50, 51] and 0.01% in primates [50–52].To further evaluate if this modest brain exposure increase was mechanism-related and to monitor an early time after injection (1 h), we performed in vivo panning of a library of anti ITM2A antibodies. A naive human antibody-phage library was enriched for ITM2A specific antibodies against recombinant protein ITM2A and the antibody-phage output was amplified and purified by PEG/NaCl purifications before injection to a single mouse. The brain was harvested at 1 and 24 h after injection and the homogenates were used for infection of *E.* *coli*. From this no hit was identified, suggesting that none of the antibody-phages were able to specifically reach the brain parenchyma. Phages are huge entities, and their barrier crossing might be more difficult than isolated antibodies. In addition, our anti-ITM2A antibody could be trapped in the vessels or recycling [53]. Alternatively, the epitope recognized by the antibody we selected for the in vivo study might not be the one leading to transcytosis. To finally conclude on the fate of anti-ITM2A antibodies after in vivo injection and their potential to enhance brain exposure, several antibodies recognizing distinct epitopes should be compared, and the antibody levels measured in both parenchyma and vessels. Luminal expression is key for a brain delivery mechanism. It would be important to analyze luminal/abluminal localization of ITM2A. This could be performed using in vivo biotinylation [54] or colocalization experiments with PGP on mouse brain sections by immunohistochemistry [55]. At this stage, we considered that the enhancement that could be obtained with ITM2A was not at the level that could be of interest for a potential application to a therapeutic project.

## Conclusions

Our work combines transcriptomic profiling leading to selecting ITM2A as a potential brain specific target, with in vivo phage panning of an anti ITM2A phage library. Our approach illustrates the complexity of such an endeavor. Beyond the technical challenge of getting access to pure human primary endothelial cells, highly expressed proteins are often down regulated when endothelial cells are grown in culture, making it difficult to study them in functional cellular models, and human-mouse cross-reactive monoclonal antibodies are necessary for validation in rodent models. In addition, potential targets might have different functions in rodents and humans, although we have no indication that this could be the case for ITM2A. ITM2A might remain a valid target for enhancing the delivery of drugs to the human brain but its validation might prove quite complex.

## Supplementary Information

**Additional file 1: Figure S1.** Clonal selection of stable HEK293 overexpressing ITM2A. After fluorescence selection, better clones were confirmed by Western Blot. Clones with higher expression and higher luminescent signal were selected. Selected clones are circled in red. **A** HEK293 ITM2A human C-Ter and Nter GFP clone selection. **B** HEK293 ITM2A murin Nter GFP clone selection. **C** HEK293 ITM2A murin C-Ter GFP clone selection. **D** HEK293 ITM2A human C-Ter HA clone selection. **E** HEK293 ITM2A human N-Ter HA and WT clone selection.

**Additional file 2: Figure S2.** Western Blot membrane of relative quantification of ITM2A protein expression in different cells type. Signal was detected by antibody anti-ITM2A AF4876 followed by HRP coupled anti-sheep antibody. Then, luminescence was quantified.

**Additional file 3: Figure S3.** Western Blot membranes of ITM2A relative quantification expression in new born mice. Signal was detected by antibody anti-ITM2A AF4876. HEK293 ITM2A mouse GFP is used as control. On the left a-tubulin T9026 detection on the right anti-ITM2A AF4876. Detection with primary antibody was followed by HRP coupled with appropriate antibody. Then, luminescence was quantified.

**Additional file 4: Figure S4.** Detection limit of mouse ITM2A by western blot in HEK293 overexpressing mITM2A. Signal is quantified with MultiGauge v3.0. Housekeeping protein is α-tubulin in orange and protein of interest is ITM2A in blue.

## Data Availability

All data generated or analyzed during this study are included in this published article and its supplementary information files.

## Abbreviations

ANOVA: Analysis of variance
BBB: Blood brain Barrier
BEC: Brain endothelial cell
BMEC: Brain microvascular endothelial cells
BSA: Bovine serum albumin
CD: Cluster of differentiation
CMV: CytoMegaloVirus
CNS: Central nervous system
COMPACT: Collaboration on the optimization of macromolecular pharmaceutical
Ct: Cycle treshold
C-Ter: C-terminal
EBM: Endothelial basal medium
ECD: Extracellular domain
ELISA: Enzyme-linked immunosorbent assay
Em: Emerald
FPKM: Fragments per kilobase million
GAPDH: GlycerAldehyde-3-phosphate deshydrogenase
GFP: Green fluorescent protein
HA: HemAgglutinin
HBSS: Hanks’ balanced salt solution
hCMEC/D3: Human cerebral microvascular endothelial cell
HEK293: Human embryonic kidney
HRP: Horseradish peroxidase
ICD: Intracellular domain
IgG: Immunoglobulin G
IMI: Innovative medicines initiative
ITM2A: Integral membrane protein 2A
LAMP1: Lysosomal-associated membrane protein 1
LC/MS: Liquid chromatography/mass spectrometry
LRP8: Low-density lipoprotein receptor-related Protein 8
mAb: Monoclonal antibody
MDCK: Madin-Darby canine kidney
MW: Molecular weight
NGS: Next generation sequencing
NHP: Non-human primate
N-Ter: N-terminal
P0D0: Passage 0 day in vitro 0
P1: Post-birth 1
Papp: Apparent permeability
PBS: Phosphate-buffered saline
PEG: Poly-ethylene glycol
PVDF: Polyvinylidene fluoride
RIPA: Radio-immunoprecipitation assay
RT: Room temperature
RTqPCR: Reverse transcription quantitative polymerase chain reaction
scFv: Single-chain variable fragment
SDS: Sodium dodecyl sulfate
SIL: Stable isotope labelling
TBST: Tween tris-buffered saline
TFRC: Transferrin receptor C
TLCK: Tosyl-l-lysyl-chloromethane hydrochloride
TM: Transmembrane domain
TNP: Trinitrophenyl
WB: Western blot
Wt: Wild type
