# Supplementary material for: Exploring ITM2A as a new potential target for brain delivery
Source: Fluids Barriers CNS. 2022 Mar 21;19:25. doi: 10.1186/s12987-022-00321-3 (PMC8935840; doi:10.1186/s12987-022-00321-3)
Supplement: Supplementary file 1 — Additional file 1: Figure S1. Clonal selection of stable HEK293 overexpressing ITM2A. After fluorescence selection, better clones were confirmed by Western Blot. Clones with higher expression and higher luminescent signal were selected. Selected clones are circled in red. A HEK293 ITM2A human C-Ter and Nter GFP clone selection. B HEK293 ITM2A murin Nter GFP clone selection. C HEK293 ITM2A murin C-Ter GFP clone selection. D HEK293 ITM2A human C-Ter HA clone selection. E HEK293 ITM2A human N-Ter HA and WT clone selection. [file 12987_2022_321_MOESM1_ESM.docx]

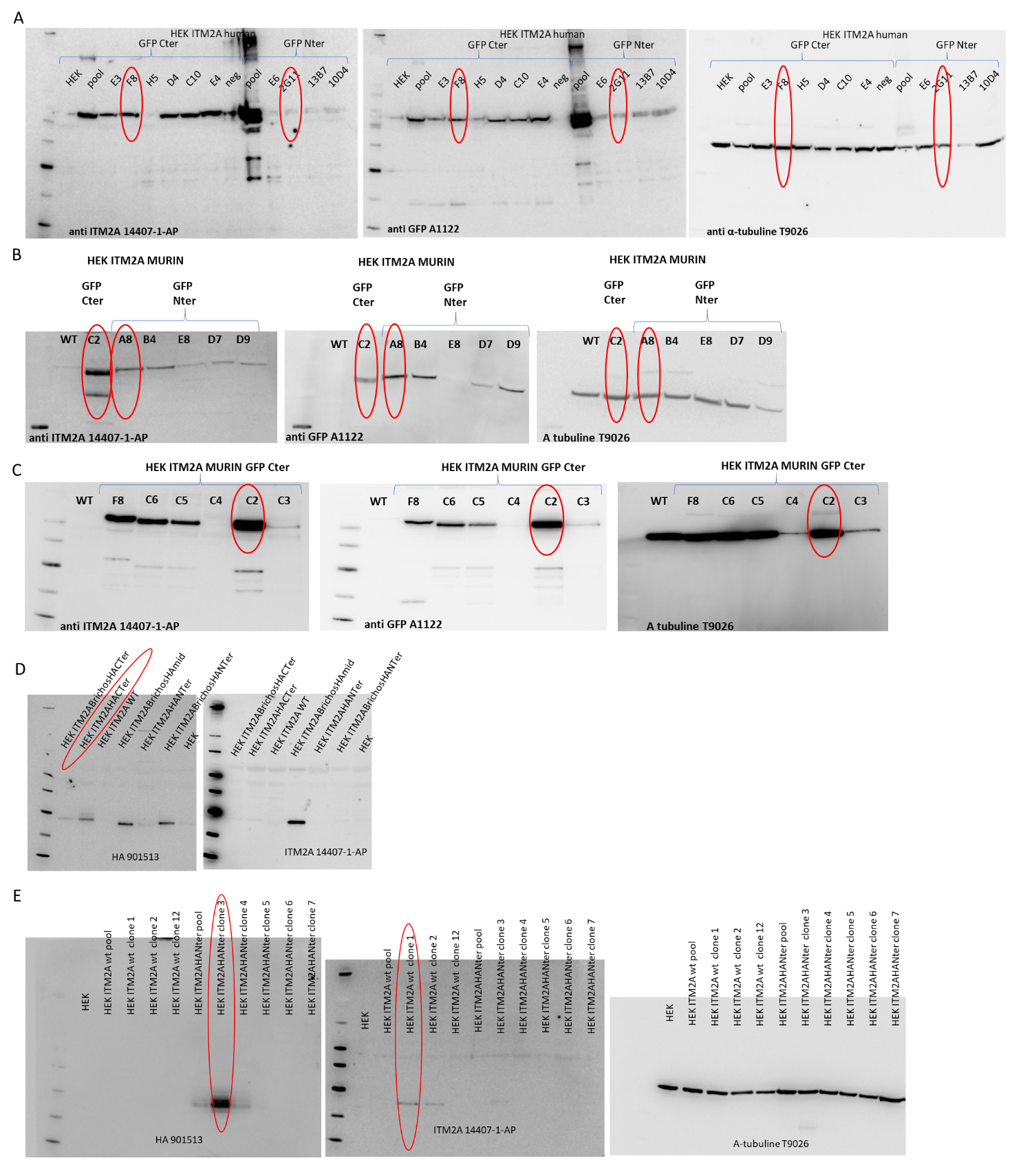


Additional file 1: Clonal selection of stable HEK293 overexpressing ITM2A.

After fluorescence selection, better clones were confirmed by Western Blot. Clones with higher expression and higher luminescent signal were selected. Selected clones are circled in red. A: HEK293 ITM2A human C-Ter and Nter GFP clone selection B: HEK293 ITM2A murin Nter GFP clone selection C: HEK293 ITM2A murin C-Ter GFP clone selection D : HEK293 ITM2A human C-Ter HA clone selection E : HEK293 ITM2A human N-Ter HA and WT clone selection
