# Supplementary material for: Exploring ITM2A as a new potential target for brain delivery
Source: Fluids Barriers CNS. 2022 Mar 21;19:25. doi: 10.1186/s12987-022-00321-3 (PMC8935840; doi:10.1186/s12987-022-00321-3)
Supplement: Supplementary file 2 — Additional file 2: Figure S2. Western Blot membrane of relative quantification of ITM2A protein expression in different cells type. Signal was detected by antibody anti-ITM2A AF4876 followed by HRP coupled anti-sheep antibody. Then, luminescence was quantified. [file 12987_2022_321_MOESM2_ESM.docx]

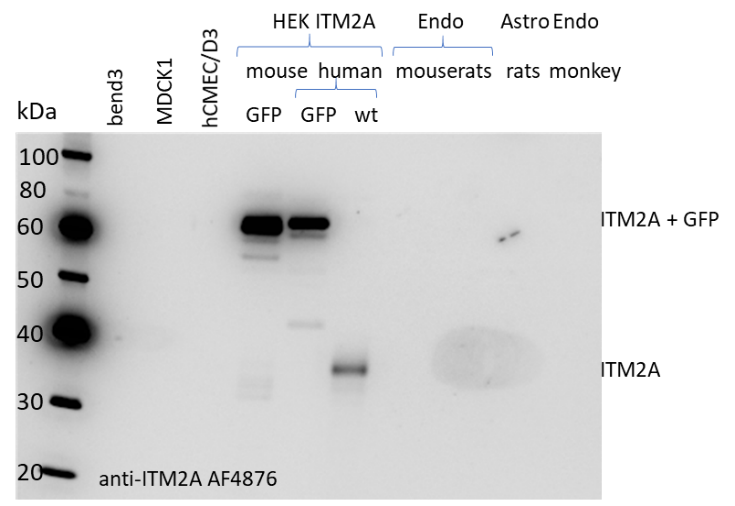


Additional file 2: Western Blot membrane of relative quantification of ITM2A protein expression in different cells type.

Signal was detected by antibody anti-ITM2A AF4876 followed by HRP coupled anti-sheep antibody. Then, luminescence was quantified.
