# Supplementary material for: Exploring ITM2A as a new potential target for brain delivery
Source: Fluids Barriers CNS. 2022 Mar 21;19:25. doi: 10.1186/s12987-022-00321-3 (PMC8935840; doi:10.1186/s12987-022-00321-3)
Supplement: Supplementary file 3 — Additional file 3: Figure S3. Western Blot membranes of ITM2A relative quantification expression in new born mice. Signal was detected by antibody anti-ITM2A AF4876. HEK293 ITM2A mouse GFP is used as control. On the left a-tubulin T9026 detection on the right anti-ITM2A AF4876. Detection with primary antibody was followed by HRP coupled with appropriate antibody. Then, luminescence was quantified. [file 12987_2022_321_MOESM3_ESM.docx]

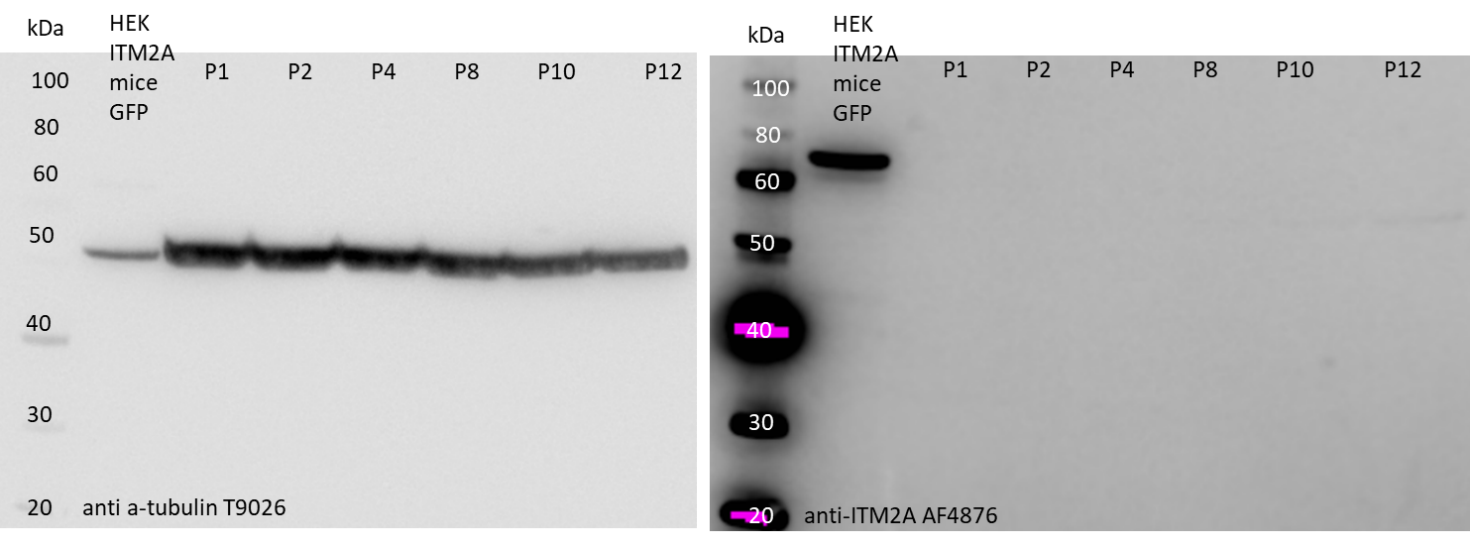


Additional file 3: Western Blot membranes of ITM2A relative quantification expression in new born mice.

Signal was detected by antibody anti-ITM2A AF4876. HEK293 ITM2A mouse GFP is used as control. On the left a-tubulin T9026 detection on the right anti-ITM2A AF4876. Detection with primary antibody was followed by HRP coupled with appropriate antibody. Then, luminescence was quantified.
