# Supplementary material for: Exploring ITM2A as a new potential target for brain delivery
Source: Fluids Barriers CNS. 2022 Mar 21;19:25. doi: 10.1186/s12987-022-00321-3 (PMC8935840; doi:10.1186/s12987-022-00321-3)
Supplement: Supplementary file 4 — Additional file 4: Figure S4. Detection limit of mouse ITM2A by western blot in HEK293 overexpressing mITM2A. Signal is quantified with MultiGauge v3.0. Housekeeping protein is α-tubulin in orange and protein of interest is ITM2A in blue. [file 12987_2022_321_MOESM4_ESM.docx]

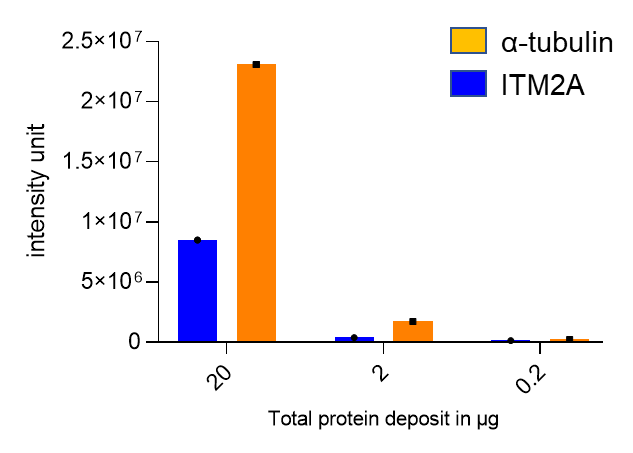


Additional file 4 Detection limit of mouse ITM2A by western blot in HEK293 overexpressing mITM2A. Signal is quantified with MultiGauge v3.0. Housekeeping protein is α-tubulin in orange and protein of interest is ITM2A in blue.
